# miR-24-3p/FGFR3 Signaling as a Novel Axis Is Involved in Epithelial-Mesenchymal Transition and Regulates Lung Adenocarcinoma Progression

**DOI:** 10.1155/2018/2834109

**Published:** 2018-04-05

**Authors:** Pengyu Jing, Nan Zhao, Nianlin Xie, Mingxiang Ye, Yong Zhang, Zhipei Zhang, Mengyang Li, Xiaofeng Lai, Jian Zhang, Zhongping Gu

**Affiliations:** ^1^Department of Thoracic Surgery, Tangdu Hospital, Fourth Military Medical University, Xi'an 710038, China; ^2^Department of Biochemistry and Molecular Biology, State Key Laboratory of Cancer Biology, Fourth Military Medical University, Xi'an 710032, China; ^3^Department of Public Health, Xi'an Medical University, Xi'an 710021, China; ^4^Department of Pulmonary Medicine, Xijing Hospital, Fourth Military Medical University, Xi'an 710032, China; ^5^Department of Hepato-Biliary and Pancreto-Splenic Surgery, Xijing Hospital, Fourth Military Medical University, Xi'an 710032, China

## Abstract

Our previous studies showed that Fibroblast growth factor receptor 3 (FGFR3) contributed to cell growth in lung cancer. However, the correlation between FGFR3 and tumor progression, coupled with the underlying mechanisms, are not fully understood. The clinical significance of FGFR3 was determined in two cohorts of clinical samples (*n* = 22, *n* = 78). A panel of biochemical assays and functional experiments was utilized to elucidate the underlying mechanisms and effects of FGFR3 and miR-24-3p on lung adenocarcinoma progression. Upregulated FGFR3 expression indicated an adverse prognosis for lung adenocarcinoma individuals and promoted metastatic potential of lung adenocarcinoma cells. Owing to the direct regulation towards FGFR3, miR-24-3p could interfere with the potential of proliferation, migration, and invasion in lung adenocarcinoma, following variations of EMT-related protein expression. As a significant marker of EMT, E-cadherin was negatively correlated with FGFR3, of which ectopic overexpression could neutralize the antitumour effects of miR-24-3p and reverse its regulatory effects on EMT markers. Taken together, these findings define a novel insight into the miR-24-3p/FGFR3 signaling axis in regulating lung adenocarcinoma progression and suggest that targeting the miR-24-3p/FGFR3 axis could be an effective and efficient way to prevent tumor progression.

## 1. Introduction

As its lethality rate ranks near the top, lung cancer is regarded as a very common malignancy [1]. More than 90% of cancer-related deaths in nonsmall cell lung cancer (NSCLC) are triggered by tumor progression, especially metastasis [1, 2]. Being a primary subtype of NSCLC, lung adenocarcinoma is liable to metastasize at early stage than lung squamous carcinoma. Accordingly, it is necessary to investigate the mechanisms related to lung adenocarcinoma metastasis. Overexpression or activation of some oncogenes could draw a dramatic shift in cancer cell performance, such as improving proliferative or metastatic potentials of tumor cells.

Fibroblast growth factor receptors (FGFRs) mediate a set of development-related pathways, such as the formation of mesoderm during the early embryonic stage and the development of multiple organs and systems. FGFR3, a highly conserved transmembrane tyrosine kinase receptor, overexpressed aberrantly in bladder [3], cervical [4], and colorectal cancer [5], suggesting that abnormal expression of FGFR3 was blamed to contribute partially to tumorigenesis. In addition, many studies indicated that FGFR3 played key roles in regulation of cellular differentiation, multiplication, apoptosis, and migration [6, 7]. Our previous study also showed that downregulation of FGFR3 (via knockdown of protein arginine methyltransferase 5 (PRMT5)) dramatically suppressed proliferation of lung tumor cells and abolished progression of xenograft tumors [8]. These studies demonstrated that FGFR3 was concerned with the tumorigenesis of lung cancer. However, the active effects and molecular mechanism of FGFR3 in lung adenocarcinoma are only partially understood and warrant further investigation.

microRNAs (miRNAs) combine the 3′-untranslated regions (3′-UTRs) of targeted mRNAs by means of complementary base pairing, leading to either degradation or translational silencing of target genes. miRNAs are involved in multiple cellular programs correlated with carcinogenesis, including differentiation, proliferation, metabolism, apoptosis, migration, and invasion [9–11]. In recent decades, dysregulated expression of miRNAs was identified as either novel biomarkers or promising therapeutic targets of human malignant tumors. Among these miRNAs, miR-24-3p is one of the most important miRNA related to the occurrence and progression of tumors, whose aberrant expression has been detected in various types of cancer, including NSCLC [12], pancreatic cancer [13], gastric carcinoma [14], acute myelogenous leukemia [15], and so forth. One research recently further demonstrated that FGFR3 was targeted by miR-24-3p in multiple myeloma [16]. Nevertheless, the exact roles of miRNA in regulating FGFR3 in lung adenocarcinoma and whether this regulation is involved in tumor progression are still in need of further investigations.

Referring to tumor progression, epithelial-mesenchymal transition (EMT) participates in multiple events involved in tumor metastasis [17]. Many transcription factors are capable of orchestrating the EMT program, and the abnormal expression of these factors always results in wildly dysregulated cell behavior. For instance, Snail [18, 19], Slug [20, 21], and Twist [22], performing as inhibitory transcription factors, directly repressed E-cadherin and Claudin-1 expression, which was essential for the establishment of tight junctions between adjacent cells [23]. However, whether the miR-24-3p/FGFR3 signaling is involved in EMT in lung adenocarcinoma remains uncertain.

In our research, we systematically analyzed FGFR3 and miR-24-3p expression in lung adenocarcinoma and identified FGFR3 as a right targeted gene of miR-24-3p. Moreover, this targeted regulation suppressed progression of lung adenocarcinoma.

## 2. Materials and Methods

### 2.1. Cell Line

Lung adenocarcinoma cells A549 and H1299 were obtained from Shanghai Institutes for Biological Sciences (SIBS) and authenticated by STR profiling. 10% fetal bovine serum (Gibco, USA) was added to RPMI-1640 medium (Corning, USA) for incubation of A549 and H1299 cells and added to DMEM medium (Corning, USA) for incubation of Beas2B and HEK293 cells.

### 2.2. Tissue Samples

Two independent sets of lung adenocarcinoma samples were from Tangdu Hospital (Xi'an, China). The first cohort included 22 freshly frozen tumor samples and paired paracancerous tissues. The second cohort of samples covered 78 lung adenocarcinoma individuals who had undergone surgical resection between 2008 and 2013. The protocol was approved by the Ethics Committee of Tangdu Hospital and conducted based on the principles established by the Declaration of Helsinki.

### 2.3. Tissue Microarrays and Immunohistochemistry (IHC)

Tissue microarrays were manufactured using 78 paired lung adenocarcinoma samples and matched adjacent tissues. The typical tissue areas were marked by hematoxylin/eosin staining. Then, the tissue cores were extracted and transferred to tissue microarrays. IHC staining was implemented according to the method described in our previous studies [8, 24]. The FGFR3 antibody was purchased from Santa Cruz Biotechnology and with a dilution of 1/50. The scoring method of FGFR3 IHC color intension was as below: negative, 0 point; weak, 1 point; moderate, 2 points; and strong, 3 points. Proportion of positive staining cells were scored by routine method (<5%, 0 point; 6%–25%, 1 point; 26%–50%, 2 points; 51%–75%, 3 points; and >75%, 4 points). These two scores were multiplied to produce total score. Lung adenocarcinoma samples with low and high levels of FGFR3 expression were stratified by the median score.

### 2.4. Lentivirus Production and Cell Transfection

FGFR3 overexpression lentivirus was purchased from GeneChem (Shanghai, China), and GV358-EGFP empty vector lentivirus was acted as negative controls (NC). H1299 cell was infected with the lentivirus supernatants and selected with 3 mg/ml puromycin to produce H1299 cell with FGFR3 stably overexpression (H1299 FGFR3) or its control cell (H1299 NC). miR-24-3p mimics (miR-24-3p), miR-24-3p inhibitor (anti-miR-24-3p), and their corresponding negative control miRNAs (miR-NC and anti-miR-NC) were obtained from GenePharma (Shanghai, China). The sequences of the miRNA mimics and inhibitors are as below:

miR-24-3p mimics:

5′-UGGCUCAGUUCAGCAGGAACAG-3′

5′-GUUCCUGCUGAACUGAGCCAUU-3′;

miR-NC mimics:

5′-UUCUCCGAACGUGUCACGUTT-3′

5′-ACGUGACACGUUCGGAGAATT-3′;

anti-miR-24-3p: 5′-CUGUUCCUGCUGAACUGAGCCA-3′;

anti-miR-NC: 5′-CAGUACUUUUGUGUAGUACAA-3′.

miRNA mimics or inhibitor (200 pmol) were transfected to cells with Lipofectamine 2000 (Invitrogen, USA), according to the method described in our previous studies [25, 26].

### 2.5. Quantitative Real-Time PCR (qRT-PCR)

RNA isolation was performed with TRIzol (Invitrogen, USA). To evaluate the miRNA expression levels, qRT-PCR was implemented with Mir-XTM miRNA First-Strand Synthesis and miRNA Quantitation Kits (Clontech, USA). To analyze FGFR3 mRNA expression, qRT-PCR was implemented with SYBR® Premix Ex Taq™ kit (Takara, China). The cycling condition was as below: 95°C for 30 sec, 95°C for 10 sec (36 cycles), 60°C for 30 sec, and 72°C for 30 sec. Expression of gene was standardized to expression amount of U6 or *β*-actin and analyzed through method 2^-*Δ*ΔCt^ [27]. Primer sequences (from 5′ to 3′) are listed as below:

miR-24-3p Forward: TGGCTCACATCAGCAGGAACA;

U6 Forward: GGAACGATACAGAGAAGATTAGC;

U6 Reverse: TGGAACGCTTCACGAATTTGCG;

FGFR3 Forward: GCCTCCTCGGAGTCCTTG;

FGFR3 Reverse: CGAAGACCAACTGCTCGTG;

*β*-actin Forward: CATGTACGTTGCTATCCAGGC;

*β*-actin Reverse: CTCCTTAATGTCACGCACGAT.

### 2.6. Western Blot

Equal amounts of protein were separated using SDS-PAGE electrophoresis and transferred to nitrocellulose membranes. After blocking with nonfatty milk, these membranes were incubated with primary antibodies as follows: pFGFR3 (Abcam, 1/1000); FGFR3 (Santa Cruz, 1/200); pAkt (CST, 1/1000); Akt (CST, 1/1000); pErk1/2 (CST, 1/2000); Erk1/2 (CST, 1/2000); Snail (CST, 1/1000); Vimentin (CST, 1/1000); E-Cadherin (CST, 1/1000); and *β*-actin (Boster, 1/2000). The membranes were then incubated with horseradish peroxidase-conjugated IgG (CST, 1/3000). Protein blot was detected using the ECL system (Millipore).

### 2.7. MTT Assays

Cells (2000 cells per well) were planted and incubated for 5 days. Cell viability was assessed at the same time on the 1st, 2nd, 3rd, 4th, and 5th day. After addition of 5 mg/ml MTT solution, cells were incubated for 4 hours and then removed the supernatant and added DMSO (150 *μ*l per well). Absorbance of each well was tested by iMark™ Microplate Absorbance Reader (Bio-Rad).

### 2.8. Cell Colony Formation Assays

Cell (200 cells per well) was reseeded and incubated. Cells were seeded in six-well plates at a density of 200 cells per well, followed by the addition of 3 ml culture medium for two weeks. Cell colonies were fixed using methanol and stained by crystal violet (0.1%). Images were acquired using microscope and the number of cell colonies was counted manually.

### 2.9. Wound Healing Assays

Cells were planted into 6-well plates. Wounds were produced manually. Photographs of the scratched wells were taken daily in same perspective, and five artificial wounds were randomly selected from each well. Then, the measured distances between the wound edges were calculated with Image-J software.

### 2.10. Transwell Assays

Transwell chambers (Corning, USA) with an 8 *μ*m polycarbonate membrane were covered or uncovered by Matrigel (BD, USA) for invasion or migration assays. Cell was suspended in 0.5 ml of free serum cell culture medium and then put into the transwell chamber. Cell culture medium containing 10% fetal bovine serum was added into the lower wells. After a 24-hour incubation, cells migrated and invaded through polycarbonate membrane were fixed and stained. Ten random fields were photographed and manually counted.

### 2.11. Dual Luciferase Reporter Assays

The wild type or mutant response fragments of miR-24-3p were cloned into the 3′-UTR of FGFR3; the entire elements were then cloned into the luciferase reporter vectors, pMIR-REPORT. The resulting recombinant plasmids were pMIR-REPORT-FGFR3 wild type (FGFR3-WT) and pMIR-REPORT-FGFR3 mutant (FGFR3-MUT). H1299 cells were cultured and cotransfected with either FGFR3-WT or FGFR3-MUT (200 ng) and either miR-24-3p or miR-NC mimics (200 pmol). After a 48-hour incubation, luciferase activity was detected with luciferase assay kits (Promega, USA). Activity of Firefly luciferase was standardized by activity of Renilla luciferase.

### 2.12. Statistical Analysis

Values were presented as the mean ± standard error. Statistical differences were performed with Student's *t*-test or analysis of variance (ANOVA) as appropriate. Overall survival was assessed by Kaplan–Meier analysis and log-rank test. Each experiment was repeated at least three times. The statistical significance was set at ^∗^*P* < 0.05, ^∗∗^*P* < 0.01, and ^∗∗∗^*P* < 0.001.

## 3. Results

### 3.1. FGFR3 Overexpression Was a Prognostic Factor and Positively Regulated Progression in Lung Adenocarcinoma

**Figure 1:**
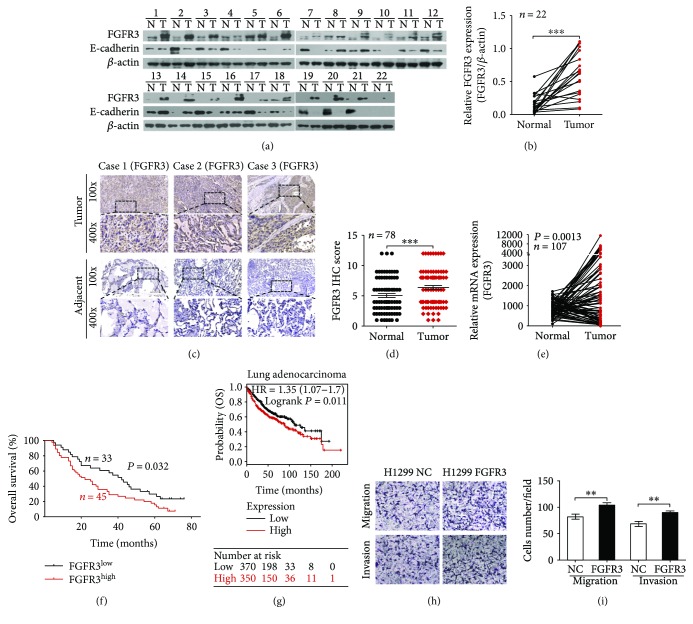
FGFR3 expression and its role in lung adenocarcinoma. (a) and (b) Western blot analysis of FGFR3 expression in tumor (T) and paired normal (N) tissues from 22 lung adenocarcinoma patients. (c) Representative IHC staining image of FGFR3 in tumors and adjacent noncancerous tissues. (d) Scatter plot of FGFR3 expression (IHC scores) in lung adenocarcinoma and adjacent noncancerous tissues from 78 patients. (e) Analysis of FGFR3 mRNA levels in NSCLC samples from the MethHC database. (f) OS of lung adenocarcinoma patients with low versus high FGFR3 expression. (g) Kaplan–Meier estimates of the cumulative survival rate based on the Kaplan–Meier plotter database. (h) and (i) Representative image of the migration and invasion assay results; the number of migratory and invasive cells is shown (*n* = 10). ^∗∗^*P* < 0.05 and ^∗∗∗^*P* < 0.01.

We measured FGFR3 expression in the initial cohort of 22 paired human lung adenocarcinoma and noncancerous tissues by Western blot. FGFR3 protein expression was dramatically higher in tumor (T) than in normal (N) samples (Figure 1(a), top panels, and Figure 1(b)). To further confirm this result, we performed IHC to score the FGFR3 expression in a second cohort of paired lung adenocarcinoma and noncancerous tissues (*n* = 78). As shown in Figure 1(c), the majority of tumor samples revealed that FGFR3 expression was greater than corresponding normal samples. Similar results of corresponding IHC scores could also be produced (Figure 1(d)). Meanwhile, individuals with high FGFR3 expression exhibited a relatively higher tendency of lymphatic metastasis (Table 1). No remarkable correlations nonetheless were discovered between FGFR3 and age, gender, TNM stage, tumor size, or distant metastasis (Table 1). In addition, we analyzed FGFR3 mRNA expression in NSCLC patients using the MethHC database (http://methhc.mbc.nctu.edu.tw/php/index.php) [28], and the results indicated that mRNA expression of FGFR3 in tumor (T) was escalated, compared with that in normal (N) samples (Figure 1(e)).

To investigate the underlying effects of FGFR3 in prognosis, we then assessed the relationship between FGFR3 protein expression and its prognosis value in lung adenocarcinoma patients. The overall survival of individuals bearing high-level FGFR3 was obviously below the patients expressing FGFR3 in a low grade. The 5-year survival rates among patients with indicated FGFR3 expression levels were 17.18% and 27.27%, respectively (Figure 1(f)). To further confirm this result, we analyzed FGFR3 expression in lung adenocarcinoma at the genomic level grounded on the database of Kaplan–Meier plotter (http://kmplot.com/analysis/) [29], which further verified our previous speculation that higher FGFR3 was closely related to poor prognosis in lung adenocarcinoma (Figure 1(g)). Collectively, our findings above indicated that FGFR3 may be a novel prognostic biomarker for the diagnosis and management of lung adenocarcinoma.

To study potential function of FGFR3 in lung adenocarcinoma progression, we constructed H1299 FGFR3 cell by infecting with corresponding lentivirus, taking H1299 NC cell as control. In accordance with our previous research, which reported that FGFR3 promoted cell growth in A549 cells [8], we observed that overexpression of FGFR3 in H1299 markedly improved the abilities to migration and invasion by transwell assays (Figures 1(h) and 1(i)). In conclusion, these findings illustrated that FGFR3 plays crucial roles in facilitating growth and metastasis in lung adenocarcinoma.

### 3.2. FGFR3 Is a Direct Target Gene of miR-24-3p

**Figure 2:**
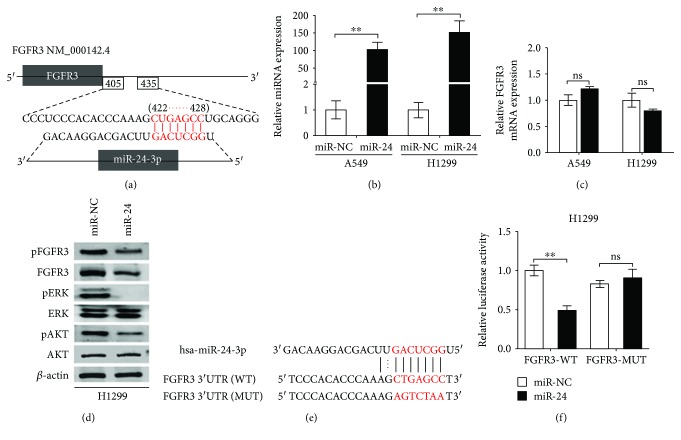
FGFR3 is a direct target of miR-24-3p. (a) miR-24-3p binding sites in 3′-UTR of FGFR3 were predicted by TargetScan. The seed region of miR-24-3p and the recognition site in the FGFR3 3′-UTR are shown in red. (b) qRT-PCR analysis of FGFR3 mRNA expression in A549 and H1299 cells transfected with either miR-NC or miR-24-3p mimics. (c) Expression of miR-24 in A549 and H1299 cells was detected by qRT-PCR. ns: not significant. (d) Cell lysates from H1299 cells that overexpressed miR-24-3p were analyzed for FGFR3, ERK1/2, and AKT content as well as the levels of the corresponding phosphorylated proteins. (e) The sequences of the WT and MUT FGFR3 3′-UTR were used for the dual luciferase reporter construct. (f) The pMIR-REPORT vector and either FGFR3-WT or FGFR3-MUT plasmids were cotransfected with miR-24-3p or miR-NC mimics in H1299 cells, after which relative luciferase activity was detected. ^∗∗^*P* < 0.01.

We next employed TargetScan computational algorithm to predict which underlying miRNAs performed targeting FGFR3 directly. Among these alternative miRNAs, miR-24-3p was chosen to be verified thoroughly in light of its frequence related to malignancies [30]. The predicted sequences between miR-24-3p and its targeted fragment within the 3′-UTR of FGFR3 were elucidated in Figure 2(a). To verify whether miR-24-3p can influence FGFR3 in lung adenocarcinoma cell lines, miR-24-3p was transfected into A549 and H1299 cells to evaluate the expression of FGFR3 in indicated groups. It found that miR-24-3p was dramatically escalated (Figure 2(b)), while there was no significant difference in mRNA of FGFR3 in both cell lines (Figure 2(c)). Surprisingly, however, FGFR3 protein was dramatically decreased in these cells with indicated treatments (Figure 2(d), second panel). These results demonstrated that the FGFR3 protein instead of FGFR3 mRNA was downregulated by miR-24-3p in lung adenocarcinoma cells, further indicating that the regulation of FGFR3 was based on miR-24-3p-mediated posttranscriptional modification.

As two major downstream signal molecules of FGFR3 [31–33], ERK and AKT were detected in H1299 cells with miR-24-3p overexpression by Western blot assay. These findings revealed that both total FGFR3 and phosphorylated FGFR3 (pFGFR3) protein were significantly decreased corresponding to changes of miR-24-3p overexpression (Figure 2(d)). Phosphorylation of ERK1/2 and AKT (pERK1/2 and pAKT) was prominently decreased in response to miR-24-3p overexpression, with no significant changes in total ERK1/2 and AKT protein (Figure 2(d)). Additionally, we investigated that miR-24-3p reduced FGFR3 protein expression was mediated by the specific interaction between miR-24-3p and the predicted targeted fragment in the 3′-UTR of FGFR3. The WT and MUT FGFR3 3′-UTR regions highlighting the miR-24-3p binding sites are shown in Figure 2(e). Besides, bioluminescence imaging assay was performed to further illustrate the potential interaction, indicating that luciferase activity of FGFR3-WT, instead of FGFR3-MUT, was dramatically reduced in H1299 cells with miR-24-3p overexpression (Figure 2(f)). These outcomes suggested that miR-24-3p suppressed FGFR3 expression through directly combining to its 3′-UTR region.

### 3.3. miR-24-3p Decreased in Lung Adenocarcinoma

**Figure 3:**
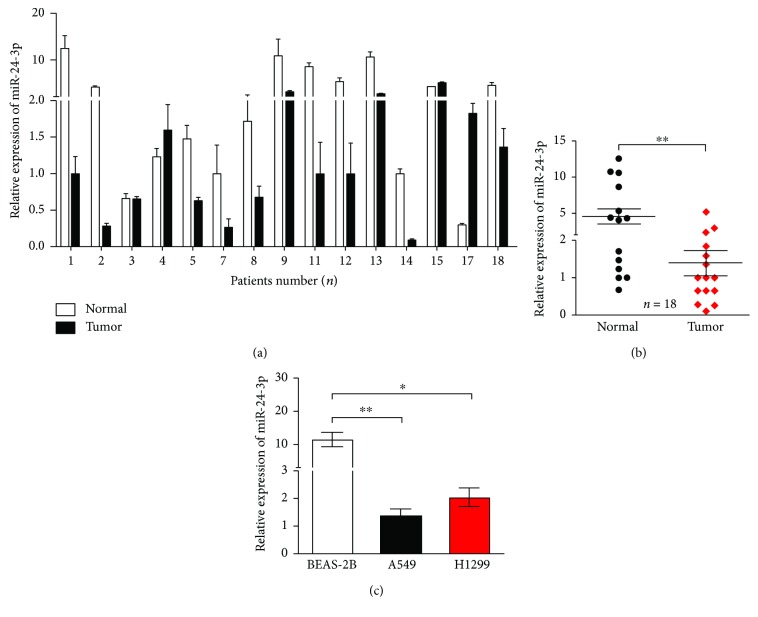
miR-24-3p is downregulated in lung adenocarcinoma tissues and cells. (a) and (b) miR-24-3p expression was significantly decreased in 18 lung adenocarcinoma tissues (Tumor), compared with the levels in normal tissues (Normal). (c) miR-24-3p expression was significantly downregulated in lung adenocarcinoma cell A549 and H1299 compared with levels in normal pulmonary epithelial cell BEAS-2B. ^∗^*P* < 0.05 and ^∗∗^*P* < 0.01.

To validate the differences of indicated miRNA in clinical samples, qRT-PCR assays were implemented to detect miR-24-3p levels in the first cohort of tissues and revealed that miR-24-3p was significantly lower in tumor samples than that in their corresponding normal samples (Figures 3(a) and 3(b)). In accordance with the results in lung cancer cell lines, miR-24-3p expression was dramatically decreased in A549 and H1299 compared with that in BEAS-2B cells (Figure 3(c)). These findings suggested that lower expression of miR-24-3p in lung cancer partially contributed to tumorigenesis and progression of lung adenocarcinoma.

### 3.4. miR-24-3p Restrained Proliferation of Lung Adenocarcinoma Cell Lines

**Figure 4:**
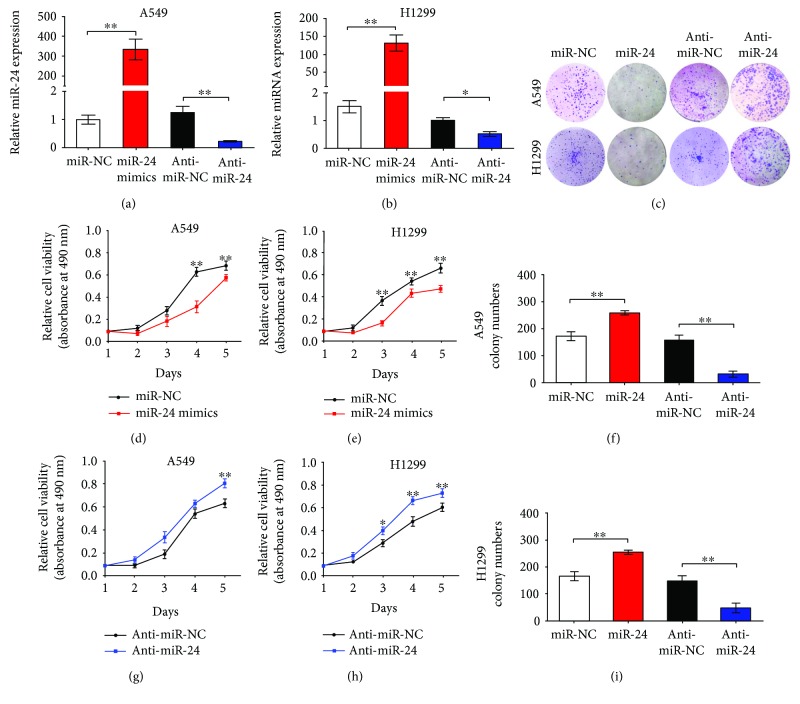
miR-24-3p suppressed the proliferation and growth of lung adenocarcinoma cells. (a) and (b) miR-24-3p expression was detected by qRT-PCR in A549 and H1299 cells transfected with miR-24-3p, anti-miR-24-3p, or respective controls. (c–f) The viability of A549 and H1299 cells transfected with miR-24-3p, anti-miR-24-3p, or their respective controls was evaluated using MTT assay. (g–i) Representative images of colony formation assays in A549 and H1299 cells transfected with miR-24-3p, anti-miR-24-3p, or respective controls (*n* = 3). ^∗^*P* < 0.05 and ^∗∗^*P* < 0.01.

We performed cell colony formation assays and MTT assays to evaluate biology effects of miR-24-3p on cellular proliferation and growth in lung adenocarcinoma. miR-24-3p expression in A549 and H1299 cell with miR-24-3p or anti-miR-24-3p overexpression was dramatically increased or decreased, respectively, compared with their corresponding NC cells (Figures 4(a) and 4(b)). Meanwhile, miR-24-3p overexpression dramatically decreased viability of A549 and H1299 cell (Figures 4(c) and 4(d)). In contrast, suppressing miR-24-3p expression with anti-miR-24-3p significantly increased proliferation potential of H1299 and A549 cells (Figures 4(e) and 4(f)). Besides, colony formation assays showed that H1299 and A549 cells with miR-24-3p overexpression generated lesser colonies than those with transfection of miR-NC as control (Figures 4(g)–4(i)), which was contrast with cells whose miR-24-3p was decreased by transfection of anti-miR-24-3p (Figures 4(g)–4(i)). Conclusions we could draw from these findings were that miR-24-3p restrained proliferation and growth of lung adenocarcinoma cell.

### 3.5. miR-24-3p Inhibited Migration and Invasion of Lung Adenocarcinoma Cell

**Figure 5:**
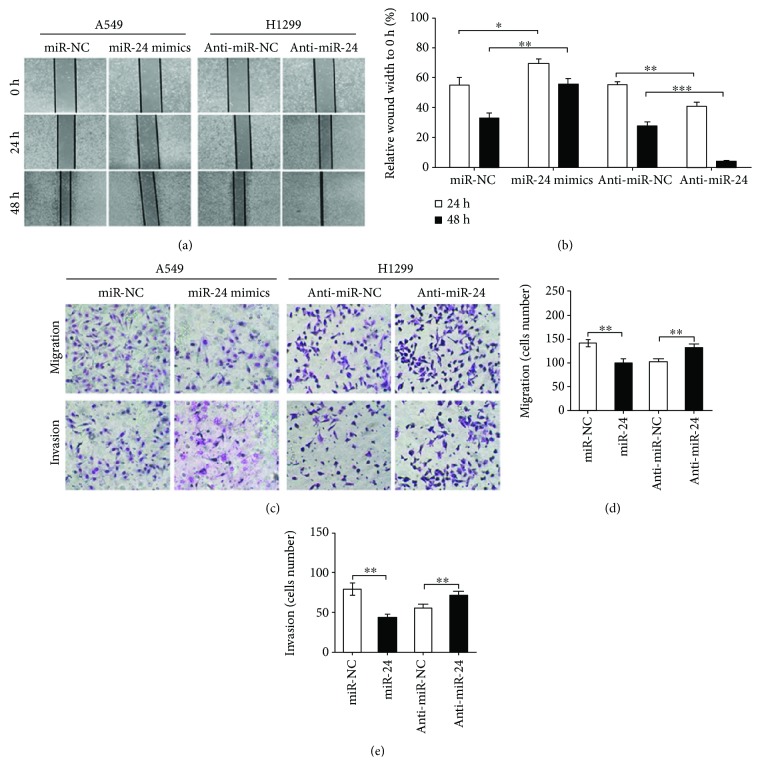
miR-24-3p suppressed the migration and invasion of lung adenocarcinoma cells. (a) and (b) miR-24-3p regulated lung adenocarcinoma cell migration (*n* = 5). A549 and H1299 cells were transfected with miR-24-3p, anti-miR-24-3p, or respective controls. (c–e) Representative images of migration and invasion assays (c). A549 cells transfected with miR-24-3p or miR-NC were cultured in transwell chambers, and the number of migratory (d) and invasive (e) cells are shown (*n* = 10). ^∗^*P* < 0.05, ^∗∗^*P* < 0.01, and ^∗∗∗^*P* < 0.001.

Due to the inhibitory roles of FGFR3 in invasion and migration of cancer cells, we then investigated whether miR-24-3p, the upstream molecule of FGFR3, affected migration and invasion of lung adenocarcinoma cells. By utilizing scratch healing assays, we found that overexpression of miR-24-3p significantly weakened the scratch healing capacity of A549 cells (Figures 5(a) and 5(b)). In addition, inhibiting miR-24-3p expression facilitated scratch wounds recombined in H1299 cells (Figures 5(a) and 5(b)). Similarly, transwell assays demonstrated the promoting-migration roles of miR-24-3p overexpression in further steps (Figures 5(c)–5(e)). In conclusion, our findings revealed that miR-24-3p possessed the ability to suppress invasion and migration in lung adenocarcinoma cell.

### 3.6. FGFR3 and EMT Signaling Were Regulated by miR-24-3p

**Figure 6:**
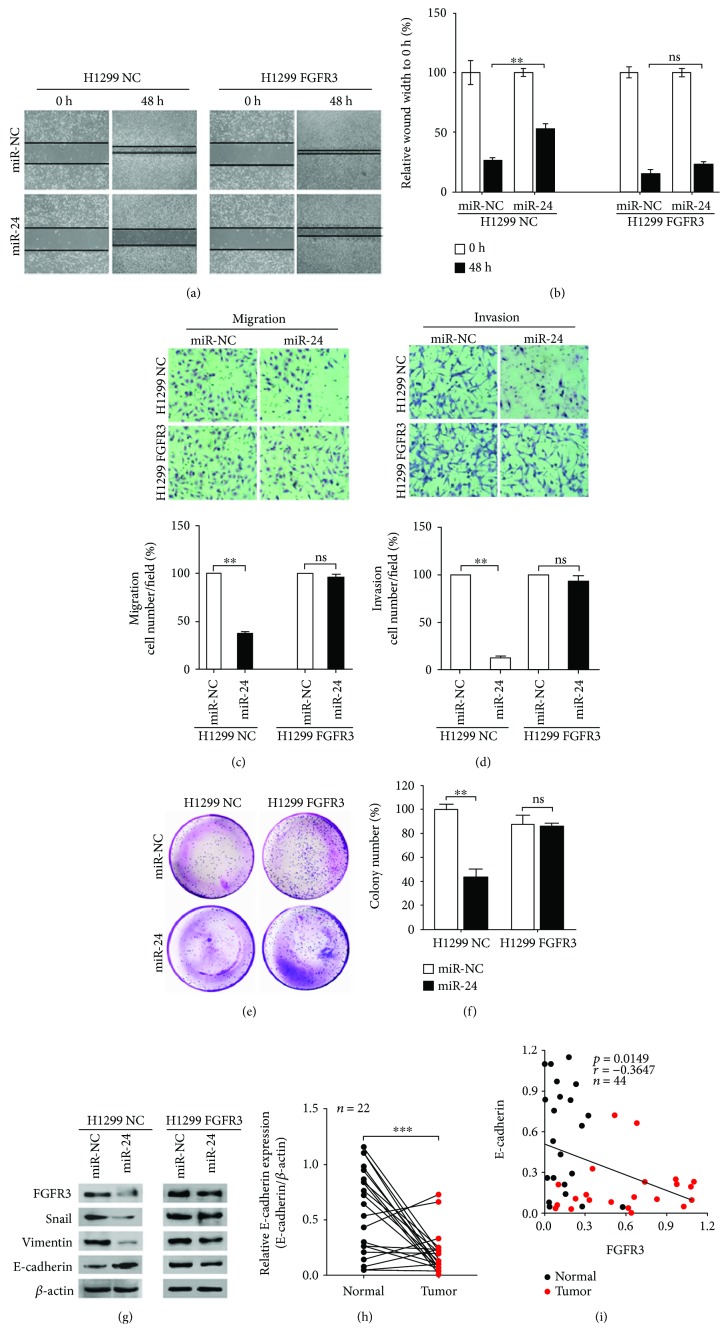
miR-24-3p inhibited growth and metastasis of lung adenocarcinoma cells and regulated EMT markers via FGFR3. (a) and (b) H1299 cells with stable overexpression of FGFR3 were transfected with miR-24-3p or miR-NC mimics, and their migration potentials were demonstrated by wound healing assays (*n* = 5). ns: not significant. (c) and (d) H1299 cells with stable overexpression of FGFR3 were transfected with miR-24-3p or miR-NC, and their migration and invasion potentials were demonstrated by the transwell assays (*n* = 5). (e) and (f) H1299 cells with stable FGFR3 overexpression were transfected with miR-24-3p or miR-NC mimics, and their proliferative ability was measured using colony formation assays. (g) H1299 cells transfected with miR-24-3p exhibited downregulated Snail and Vimentin expression and upregulated E-cadherin expression. H1299 cells with stable FGFR3 overexpression and transfection with miR-24-3p showed no significant alterations in Snail, Vimentin, and E-cadherin expression. (h) Western blot analysis of E-cadherin expression in lung adenocarcinoma tissues related to Figure 1(a). (i) Correlation analysis comparing FGFR3 and E-cadherin expression in 22 pairs of N and T samples corresponding to the cohort related to Figure 1(a). ^∗∗^*P* < 0.01 and ^∗∗∗^*P* < 0.001.

In light of the inhibitory effect of miR-24-3p in impairing processes of proliferation, migration, and invasion in lung adenocarcinoma and the direct interaction between miR-24-3p and FGFR3, we next explored whether FGFR3 took effects in miR-24-3p-mediated lung adenocarcinoma progression. After the transfection of miR-24-3p, H1299 FGFR3 cells were liable to recover the scratch wounds compared with H1299 NC cells (Figures 6(a) and 6(b)). Similar results could be found by transwell assays (Figures 6(c) and 6(d)). Furthermore, the inhibition of miR-24-3p on proliferation was detected in H1299 NC cells rather than H1299 FGFR3 cells (Figures 6(e) and 6(f)). These findings indicated that FGFR3 overexpression was able to restore the antimetastatic effects of miR-24-3p in lung adenocarcinoma cells and performed as a significant mediator in miR-24-3p regulating proliferation, invasion, and migration of lung adenocarcinoma cell.

What is noteworthy is that as an important step to promote metastasis [17], EMT process was involved in FGFR3-mediated cancer metastasis by means of regulation of transcription factors, such as Snail [34, 35]. Therefore, we evaluated whether miR-24-3p negatively regulated FGFR3 in an EMT-based pathway. Our findings revealed that protein levels of FGFR3, Snail, and Vimentin were markedly downregulated in response to miR-24-3p overexpression in H1299 NC cell (Figure 6(g), left), while these reductions were not found in H1299 FGFR3 cell (Figure 6(g), right). Likewise, under the stress of miR-24-3p overexpression, E-cadherin protein was obviously increased in H1299 NC cells but not affected in H1299 FGFR3 cells (Figure 6(g)). These findings suggested that miR-24-3p had the potential to regulate the expression of EMT markers (Snail, Vimentin, and E-cadherin) in H1299 cells and that these regulations were modulated by FGFR3. Moreover, FGFR3 overexpression could reverse the effect of miR-24-3p on these EMT markers in lung adenocarcinoma cells.

In addition, we detected E-cadherin protein expression in the first cohort of 22 paired clinical samples by Western blot. With a reduction of E-cadherin protein in tumor samples compared with the normal (Figure 1(a), middle panels, and Figure 6(h)), the expression of FGFR3 and E-cadherin exhibited a negative correlation (Figure 6(i)), indicating that miR-24-3p-mediated FGFR3 signal facilitated metastasis in an EMT-related manner.

## 4. Discussion

Cell proliferation and metastasis are the primary characteristics of tumor progression, which is one of primary contributions to cancer-related deaths. As a kind of easy-to-metastasized metastatic cancer, lung adenocarcinoma exhibits multiple and heterogeneous genomic variations and usually features a poor prognosis. Accordingly, it is necessary to investigate the mechanisms related to tumor progression and to develop novel individualized treatments based on these mechanisms. In accordance with our previous study, where FGFR3 was regarded as an oncogene that promoted lung cancer cell growth [8], we now revealed a novel metastasis mechanism of lung adenocarcinoma by that FGFR3 was negatively affected by miR-24-3p and possessed a negative correlation with E-cadherin protein, enabling cancer cell metastasis by the regulation of EMT-related proteins. More importantly, clinical evidences in this study also confirmed that FGFR3 overexpression predicted adverse clinical outcomes in patients with lung adenocarcinoma.

FGFR3 is involved in cell proliferation, differentiation, apoptosis, migration, and metastasis [6, 7]. Studies about multiple myeloma illustrated that regarded as an oncogene, FGFR3 promoted occurrence and development of tumors [36] and was escalated in several other types of tumor, indicating its negative effects in cancer pathogenesis [3–5]. Inhibiting the activity of FGFR3 by PD173074 and/or SU5402, taken as an example, resulted in cell death of multiple myeloma [37]. In addition, FGFR3 was upregulated in approximately 40% of invasive bladder tumors; this observation also implied that FGFR3 played an oncogenic role in bladder carcinoma [38]. Furthermore, tumor-specific mutations and upregulation of FGFR3 mRNA were found in multiple myeloma, bladder carcinoma, cervical cancer, and colon carcinoma [3–5, 36, 39, 40]. In accordance with the studies above, we confirmed that FGFR3 performed as an oncogene, upregulated in both tissues and cells of lung adenocarcinoma. Overexpression of FGFR3 not only significantly promoted proliferation but also obviously facilitated cell invasion and migration in lung adenocarcinoma. Furthermore, our results suggested that FGFR3 overexpression was related to the worse outcome of individuals with lung adenocarcinoma. Likewise, the Kaplan–Meier plotter database also revealed that higher levels of FGFR3 were closely related to worse prognosis in lung adenocarcinoma individuals.

The involvement of miRNAs in cancer progression by regulating a sort of cell programs, including differentiation, metabolism, growth, metastasis, and drug sensitivity, has been thoroughly discussed [9, 10, 41]. Aberrant expression of miRNAs is frequently identified as a hallmark of cancer. miR-24-3p has been demonstrated to participate in the pathogenesis of multiple myeloma [16], acute myeloid leukemia [42], nasopharyngeal carcinoma [43], and breast cancer [44] in a quite different manner. In acute myeloid leukemia and glioblastoma, miR-24-3p promoted cell proliferation and metastasis by decreasing MAPK phosphatase-7 and ST7L expression [42, 45]. In nasopharyngeal carcinoma and gastric cancer, however, it was identified as an antioncogene and was shown to target FSCN1 and RegIV [43, 46]. Herein, we verified that miR-24-3p exerted an antioncogene effect in the occurrence and development of lung adenocarcinoma. Furthermore, downregulation of miR-24-3p was partially generated by the upregulation of FGFR3 in lung adenocarcinoma.

Recently, a study, concerning the pathogenesis of multiple myeloma, indicated that FGFR3 was regarded as a targeted gene of miR-24-3p [16]. Besides, it was verified that miR-24-3p was related to EMT program. For example, miR-24-3p managed expression of E-cadherin, ZO-1, N-cadherin, and Slug by direct targeting Net1A in breast cancer [47]. These findings demonstrated that miR-24-3p had a direct interaction with FGFR3, resulting in an EMT-related progress. Similarly, we confirmed that, in lung adenocarcinoma, miR-24-3p suppressed the expression of FGFR3 as well as some EMT-associated factors, including Snail, E-cadherin, and Vimentin. In conclusion, we demonstrated that miR-24-3p/FGFR3 axis managed the occurrence and development of lung adenocarcinoma in an EMT-related manner.

## 5. Conclusions

In summary, this study demonstrated that serving as an antioncogene, overexpression or silencing of miR-24-3p resulted in obvious variations in the viability, growth, and metastasis of lung adenocarcinoma cells. Besides, FGFR3 was directly targeted by miR-24-3p and upregulation of FGFR3 was related to worse prognosis in patients with lung adenocarcinoma. These results indicated a potential prometastatic mechanism by which miR-24-3p-mediated posttranscriptional regulation of FGFR3 resulted in the accumulation of FGFR3 and, consequently, promoted EMT. Targeting the miR-24-3p/FGFR3 axis provides a new approach to prevent the progression of lung adenocarcinoma in clinic.

## Figures and Tables

**Table 1:** Relationship between FGFR3 expression and clinical characteristics in 78 lung adenocarcinoma patients.

Clinicopathological features	*n*	Percent (%)	FGFR3 expression		*P*
			Low (*n* = 33)	High (*n* = 45)	
Gender					
Male	34	43.59	16	18	0.772
Female	44	56.41	17	27	
Age (years)					
≤60	33	42.31	13	20	0.461
>60	45	57.69	20	25	
Tumor size					
T1 and T2	27	34.62	11	16	0.149
T3 and T4	51	65.38	22	29	
TNM stage					
I and II	23	29.49	13	10	0.081
III and IV	55	70.51	20	35	
Lymphatic metastasis					
Negative	46	58.97	24	22	0. 041
Positive	32	41.03	9	23	
Distant metastasis					
M0	76	97.44	32	44	0.684
M1	2	2.56	1	1	
